# Immune Cells Localize to Sites of Corneal Erosions in C57BL/6 Mice

**DOI:** 10.3390/biom13071059

**Published:** 2023-06-29

**Authors:** Phuong M. Le, Sonali Pal-Ghosh, A. Sue Menko, Mary Ann Stepp

**Affiliations:** 1 Department of Pathology and Genomic Medicine, Thomas Jefferson University, Philadelphia, PA 19107, USA; phuong.le@students.jefferson.edu (P.M.L.); sue.menko@jefferson.edu (A.S.M.); 2 Department of Anatomy and Cell Biology, School of Medicine and Health Sciences, George Washington University, Washington, DC 20037, USA; spghosh@gwu.edu; 3 Department of Ophthalmology, School of Medicine and Health Sciences, George Washington University, Washington, DC 20037, USA

**Keywords:** cornea, mouse, debridement, erosion, recurrent, immune cells, macrophage, neutrophil

## Abstract

Recurrent epithelial erosions develop in the cornea due to prior injury or genetic predisposition. Studies of recurrent erosions in animal models allow us to gain insight into how erosions form and are resolved. While slowing corneal epithelial cell migration and reducing their proliferation following treatment with mitomycin C reduce erosion formation in mice after sterile debridement injury, additional factors have been identified related to cytokine expression and immune cell activation. The relationship between recruitment of immune cells to the region of the cornea where erosions form and their potential roles in erosion formation and/or erosion repair remains unexplored in the C57BL/6 mouse recurrent erosion model. Here, high resolution imaging of mouse corneas was performed at D1, D7, and D28 after dulled-blade debridement injury in C57BL/6 mice. Around 50% of these mice have frank corneal erosions at D28 after wounding. A detailed assessment of corneas revealed the involvement of M2 macrophages in both frank and developing erosions at early stages of their formation.

## 1. Introduction

Recurrent epithelial erosions are painful and often difficult to treat. They develop due to genetic predisposition, as seen in patients with diabetes [1] and mutations in hemidesmosomal proteins [2], or following prior injury to the cornea [3,4]. Treatments aim to improve epithelial–stromal adhesion and promote basement membrane reassembly [5]. These outcomes can often be achieved by clearing debris at the erosion site and allowing the epithelium to regenerate over Bowman’s membrane. If this approach fails and erosion occurs again, phototherapeutic keratectomy with stromal puncture is often used [3].

Studies of recurrent erosions in animal models have allowed us to gain insight into how erosions form and to develop improved treatments [6,7,8,9,10,11]. While the mouse cornea lacks a thick Bowman’s layer under its epithelial basement membrane (EBM), studies using the mouse cornea have, nevertheless, shown how important the interaction between corneal epithelial cells and the basement membrane is in re-establishing homeostasis after corneal injury [6,7].

When the corneal epithelium is removed, ocular homeostasis is temporarily destroyed. The severing of the dense network of intraepithelial corneal sensory nerves results in neuronal signals being sent to the brain, which initiates an injury response whose aim is to preserve vision by protecting the cornea, lens, and retina from the type of excessive inflammation that leads to a so-called “cytokine storm” and loss of visual function. In the cornea, water and tear proteins, lipids, and other molecules enter the stroma causing stromal swelling and activation of the corneal endothelial cell pumps. Blood vessels located in the stroma below the limbus become leaky and allow immune cells to migrate into the stroma from the corneal periphery. Dendritic cells located within the basal cell layer of epithelial cells adjacent to the wound site are activated in response to neuronal signals and migrate to the draining lymph nodes.

To repair the injury, corneal epithelial cells disassemble hemidesmosomes that link them to the underlying basement membrane and migrate rapidly as a sheet to cover the exposed stroma. In the mouse, re-epithelialization after a 2 mm wound is typically completed within 24–36 h. During re-epithelialization, large numbers of immune cells accumulate underneath the leading edge and within the stroma that is left exposed at the center of the wound. They phagocytose debris and secrete cytokines and other growth factors that support corneal epithelial cell migration and prevent premature hemidesmosome reassembly. Regeneration of tissue integrity takes several weeks as the hemidesmosomes and basement membrane are reassembled.

Simple debridement injuries to the mouse cornea using a dulled blade (DB) leave the basal surface of the epithelial basal cells behind, which is attached to the EBM by hemidesmosomes containing α6β4 integrin; a rotating burr (RB) is used to remove epithelial debris, along with the EBM at the wound site [12]. The removal of the EBM with the rotating burr reduces erosion formation D28 after injury via a mechanism that alters the number and types of MMP9+ immune cells found at the wound site [13]. By 6 h after injury there are more monocytes in the DB corneal stroma. By 18 h after injury, the number of polymorphonuclear leukocytes (PMNs) and monocytes increase in the corneal stroma but there are no differences between DB and RB. By D7, the number of PMNs and monocytes present in DB and RB wounded corneas are similar to one another and to controls.

However, by D28, a time point when erosions form in ~50% of the corneas, there is a significant increase in the number of macrophages at D28 in DB compared to RB and controls. By leaving the basal membranes of the epithelial cells on the stroma, DB wounds force migrating corneal epithelial cells and immune cells to clear the debris to allow epithelial cells to adhere to the stroma. MMP9+ immune cells are present at erosion sites when and where the extracellular domain of β4 integrin is cleaved [14]. β4 integrin partners with α6 integrin to form the α6β4 heterodimer which mediates epithelial cell migration during wound healing and hemidesmosome reassembly after re-epithelialization is complete [12].

The studies cited above use BALB/c mice and implicate macrophages in erosion formation after dulled-blade wounding. BALB/c and C57BL/6 mice regulate their responses to corneal injuries made using chemical or infectious agents differently. C57BL/6 mice respond via mechanisms referred to as Th1-type and BALB/c mice via mechanisms dominated by a Th2 response [15,16]. Compared to Th2 responses, Th1 responses lead to recruitment of more activated immune cells and increase the risk of corneal perforation. Strain-specific immune responses to what are often referred to as sterile injuries, like the debridement injury model, have been less well characterized. While one study has shown that corneal re-epithelialization rates in C57BL/6 mice are faster than BALB/c mice and both strains develop a similar number of erosions at D28 after 1.5 mm dulled-blade debridement wounds [6], the immune responses of C57BL/6 mice after debridement at erosion sites have not been assessed. Here, we use high-resolution imaging of the C57BL/6 mouse cornea to evaluate the immune cell responses to debridement injury with a special emphasis on the events that take place at erosion sites.

## 2. Materials and Methods

### 2.1. Corneal Wound Healing

All animal studies performed were approved by and comply with the relevant guidelines of the George Washington University Medical Center Institutional Animal Care and Use Committee (IACUC) and the Thomas Jefferson University IACUC. In addition, they comply with the Association for Research in Vision and Ophthalmology (ARVO) Statement for the Use of Animals in Vision and Ophthalmic Research (https://www.arvo.org/About/policies/statement-for-the-use-of-animals-in-ophthalmic-and-vision-research, accessed on 15 May 2023). For all wounding experiments, 8–10-week-old male C57BL/6J mice were obtained from Charles River (Frederick MD). We realize that hormones play key roles in mediating corneal pathology in dry eye diseases [17,18] and that female and male mice may respond to injury differently. Because virgin female mice have smaller corneas than male mice of the same age, it is not possible to directly compare wound closure in both sexes simultaneously since the same size wound removes a different % of the epithelium on the corneal surface in small compared to large eyes. Debridement wounding was performed bilaterally as described previously [19]. In brief, mice were anesthetized with ketamine/xylazine and a topical anesthetic was applied to the ocular surface. A trephine was used to demarcate an area of 2.5 mm on the corneal epithelium and the epithelial cells within this area were removed using a dulled blade. After wounding, erythromycin ophthalmic ointment was applied to the injured cornea and mice were allowed to recover and sacrificed at 1-, 7-, or 28-days post wounding.

### 2.2. Immunofluorescence

Whole mouse eyes were removed immediately after euthanasia at day 1-, 7-, and 28-days post debridement wounding and fixed for 24 h at 4 °C in 3.7% of paraformaldehyde (Electron Microscopy Sciences, Hatfield, PA, USA), followed by 2 washes of DPBS with calcium and magnesium (Corning, Corning, PA, USA) at room temperature. Fixed eyes were then cryoprotected in 30% of sucrose in Mg++/Ca++ containing DPBS overnight at 4 °C. After cryoprotection, whole eyes were embedded in disposable molds filled with Polyfreeze Tissue Freezing Media Red (Polysciences, Warrington, PA, USA) and frozen in a dry ice/ethanol freezing bath before storing in −80 °C freezer for a minimum of 24 h. The frozen samples were cut with a Microm HM 550 Cryostat into 12–16 μm thick sections. For co-labeling involving a combination of pre-conjugated and unconjugated primary antibodies, whole eye cryosections were permeabilized with 0.5% Triton-X100 in Mg++/Ca++ containing DPBS for 1 h and blocked with 1% bovine serum albumin (BSA) (Fisher Scientific, Hampton, VA, USA) and 5% goat/donkey serum (Jackson ImmunoResearch Laboratories, West Grove, PA, USA) in DPBS containing 0.5% Triton-X100 for 1 h. After blocking, sections were stained with unconjugated primary antibodies for 2.5 h at 37 °C, washed twice with DPBS, then incubated for 1 h at 37 °C with secondary antibody (Jackson ImmunoResearch Laboratories) diluted in block buffer. After washing off the secondary antibody twice with DPBS, sections were stained with pre-conjugated primary antibodies with or without Alexa Fluor 647-labeled phalloidin (#A22287, Invitrogen, Waltham, MA, USA) in block buffer containing 1% BSA and 0.5% Triton-X100 for 2.5 h at 37 °C. If one of the pre-conjugated primary antibodies has the same host species as the unconjugated ones, an additional block with buffer containing 1% BSA, 10% of normal serum from the shared host and 0.5% Triton-X100 was performed for 1 h at room temperature before staining with pre-conjugated antibodies. Rat (#012-000-120) and rabbit (#011-000-120) serum used were purchased from Jackson ImmunoResearch Laboratories. For immunolabeling with pre-conjugated primary antibodies only, sections were blocked with 1% BSA in DPBS containing 0.25% Triton-X100 for 30min at room temperature before staining with primary antibodies for 2.5 h at 37 °C. Nuclei were labeled with DAPI (#422801, Biolegend, San Diego, CA, USA) for 30min before sections were washed and mounted in Prolong Diamond Antifade Mountant (#P36970, Invitrogen).

The following pre-conjugated primary antibodies were used for immunolabeling: β2 integrin (clone M18/2, 1:50), CD3 (clone 17A2, 1:100), CD4 (clone RM4-5, 1:100), CD8b (clone YTS156.7.7, 1:100), CD68 (clone FA-11, 1:50), CD206 (clone C068C2, 1:50), F4/80 (clone BM8, 1:50), and LY6G (clone 1A8, 1:50) from Biolegend; CD11b (clone M1/70, Abcam, 1:200); Arginase1 (clone D4E3M, 1:50), and iNOS (clone D6B6S, 1:50) from Cell Signaling, Danvers, MA, USA; and GR1 (clone RB6-8C5, Stem Cell Technologies, Vancouver, BC, Canada, 1:50). Unconjugated antibodies included CD49f/α6 integrin (clone GoH3, BD Pharmingen, Franklin Lakes, NJ, USA, 1:100), LY6C (clone ER-MP20, Invitrogen, 1:100), myeloperoxidase (AF3667, R&D Systems, Minneapolis, USA, 1:100), βIII tubulin (ab18207, Abcam, Waltham, MA, USA, 1:100), and Laminin 332 (J18, gift Kevin Hamill, Jonathan Jones) [20]. Details regarding the primary and secondary antibodies used in these studies can be found in Table 1 and Table 2.

### 2.3. Confocal Image Analysis and Imaris 3D Surface Rendering

Immunolabeled cryosections were imaged using a Zeiss LSM800 confocal microscope equipped with Zeiss Plan-Apochromat 40X/1.3 Oil objective. 12–15 µm Z-stacks with 0.33 or 0.5 µm optical sections were collected and analyzed using Zeiss Zen software (version 3.3). All projections were generated using the pixel of XY planes with maximum intensity along *z*-axis, if not otherwise specified. When indicated, 3D surface reconstruction was performed using Imaris software (version 9.7) 3D View tool as described previously [21]. A 3D surface structure was generated from the entire confocal Z-stack at its original magnification and can be rotated and enlarged to view at higher zooms without distortion.

### 2.4. Statistical Analysis

Statistical significance was determined by *t*-test, one-way ANOVA with Tukey post-test or Kruskal–Wallis rank test, where appropriate, for quantification of immune infiltrate and nerve density. All quantitative data were analyzed using GraphPad Prism 9.0.1 (GraphPad Software, Inc., San Diego, CA, USA) and presented as either mean ± standard error of the mean or violin plot. Adjusted *p*-value < 0.05 was considered statistically significant, with * *p* < 0.05, *** *p* < 0.001, and **** *p* < 0.0001.

## 3. Results

### 3.1. Immune Cell Phenotypes during Re-Epithelialization

**Figure 1:**
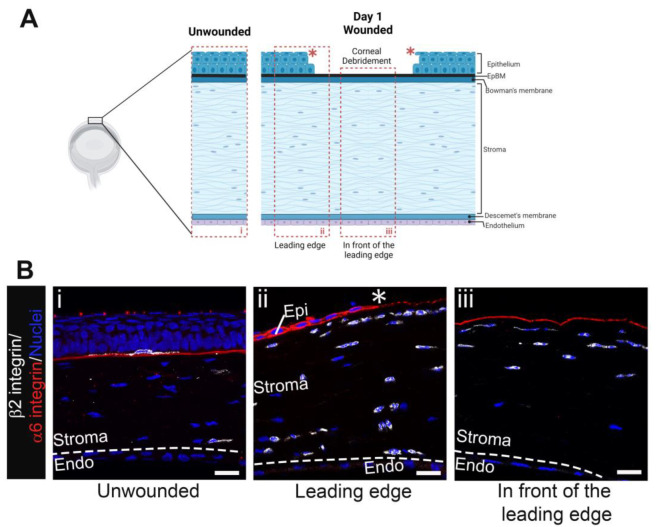
Immune cells accumulate beneath the leading edge and the region of open wound by D1 post debridement wounding. (**A**) Schematic of unwounded (**i**) and debrided cornea at D1 post wounding with boxed areas indicating regions captured in (**B**), the leading edge (**ii**), and the area in front of the leading edge (**iii**). Model created with BioRender (https://www.biorender.com/). (**Bi**) shows a β2 integrin+ (white) resident immune cell with dendritic morphology under the normally stratified corneal epithelium and occasional leukocytes in the stroma whereas (**Bii**,**Biii**) show an influx of leukocytes into the stroma beneath the intact epithelial basement membrane (EpBM) within which the extracellular domain of α6 integrin (red) left behind by the dulled blade is embedded. Nuclei were stained with DAPI (blue). Asterisk (*) and dashed line represent the wound edge and Descemet’s membrane, respectively. Studies are representative of four eyes examined. (**Bi**) 5 µm projection and (**Bii**,**Biii**) single focal plane. Mag bars: 20 µm. Endo corneal endothelium, Epi corneal epithelium.

Figure 1A shows a schematic of the mouse cornea debridement model used in these studies and highlights the two sites imaged at D1 post wounding by confocal microscopy, the leading wound edge migrating from left to right labeled ii and the center of the wound where the stroma is exposed directly to the tear film labeled iii. A snapshot of the unwounded cornea labeled i is also included for comparison. The data presented in this manuscript are representative of those obtained from four corneas at each time point indicated. Tissue sections of mouse eyes, both unwounded and at 1-day post-cornea wounding, were labeled with an antibody against the extracellular domain of α6 integrin, an epithelial protein present in hemidesmosomes which functions during epithelial cell migration, and an antibody to β2 integrin (CD18), a protein universally expressed in immune cells. Figure 1(Bi) shows continuous α6 integrin under the stratified corneal epithelium with dendritic cells occasionally identified among the basal cells, as expected. In Figure 1(Bii), α6 integrin is seen in the migrating epithelial leading edge. In front of the leading edge, α6 integrin staining is patchy. We have shown previously that α6 integrin and other basement membrane proteins are degraded by MMP9 that is produced by immune cells in the stroma. The debrided stroma shown in Figure 1(Biii) is positive for α6 integrin because the dulled-blade wound model leaves α6β4 integrin hemidesmosomes embedded in the basement membrane [13]. The uniformity of the α6 integrin staining in Figure 1(Biii) indicates that the basement membrane was not removed during debridement.

The β2 integrin staining in Figure 1(Bii) shows that at D1 post wounding immune cells were located immediately beneath and in front of the leading edge in the anterior and posterior stroma close to Descemet’s membrane and the corneal endothelial cell layer. The β2 integrin staining in Figure 1(Biii) shows immune cells in the anterior third of the corneal stroma, but the posterior stroma is devoid of immune cells. The β2 integrin+ cells closest to the α6 integrin+ basement membrane were elongated and had their axes oriented parallel to the basement membrane; they often appeared as chains consisting of one or more immune cells. This morphology was observed both beneath the leading edge (Figure 1(Bii)) and at the cornea center where the stroma is exposed directly to the tear film (Figure 1(Biii)). By contrast, the immune cells located furthest from the basement membrane were more rounded and appeared shorter.

**Figure 2:**
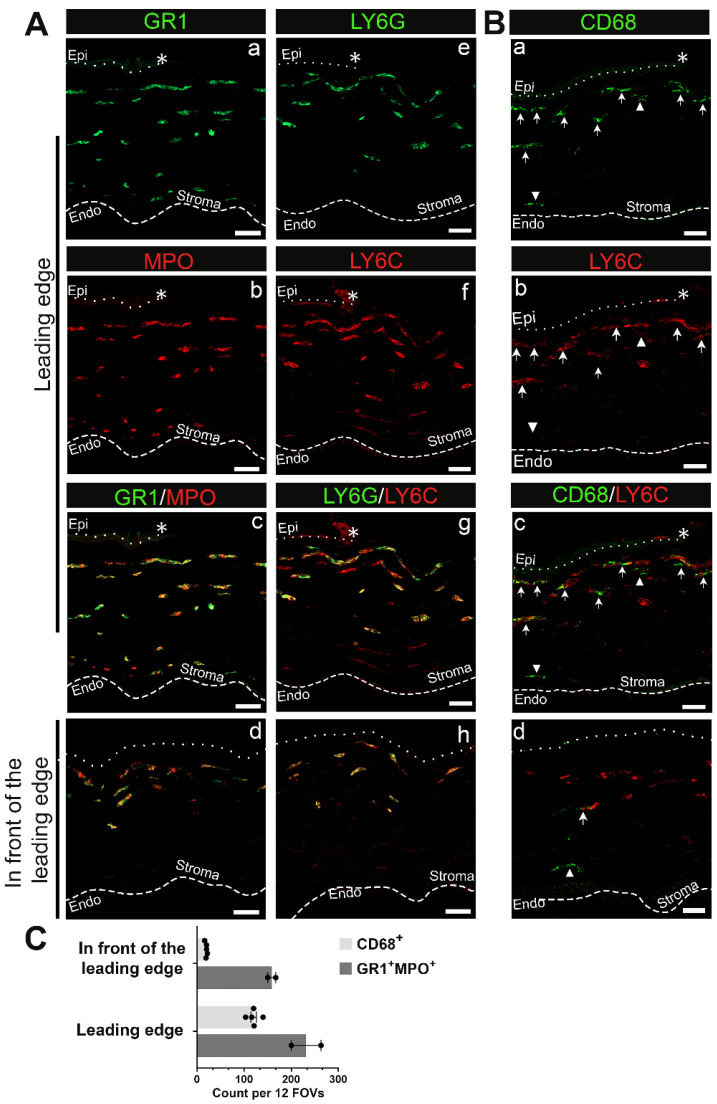
Neutrophils and to a lesser extent monocyte-macrophage lineage cells migrate into the stroma of the central cornea D1 post debridement wounding. (**A**) Representative images of neutrophils expressing GR1 and LY6G in green and LY6C and MPO in red present beneath both the leading edge (**a**–**c**,**e**–**g**) and the area in front of the leading edge (**d**,**h**). Please note that anti-GR1 recognizes a shared epitope of LY6C and LY6G. (**B**) Representative images of CD68+ monocyte-macrophage lineage cells that can be either LY6C− (arrowhead) or LY6C+ (arrow) beneath the leading edge (**a**–**c**) and in front of the leading edge (**d**). (**C**) Quantification of GR1+MPO+ and CD68+ leukocytes across the corneal stroma. Each datapoint represents the number of leukocytes of indicated subset identified in 12 fields of views (FOVs) from 3 sections per eye (n = 2 for GR1+MPO+ and n = 5 for CD68+). Asterisk (*), dotted line, and dashed line represent the wound edge, EpBM, and Descemet’s membrane, respectively. Single-channel panels for images captured in the region in front of the leading edge can be found in Supplementary Figure S2. (**A**) 3 µm projection, (**B**) 10 µm projection for the leading edge and 5 µm projection for the region in front of the leading edge. Mag bars: 20 µm. Endo corneal endothelium and epi corneal epithelium.

The different types of immune cells present cannot be distinguished by β2 integrin staining. Additional antibodies need to be used to determine the different types of immune cells present. Initial innate responses to an injury are typically dominated by neutrophils. To further characterize the types of immune cells observed in the cornea, cryosections of the mouse eyes were labeled with antibodies against MPO, GR1, Ly6G, and Ly6C. In Figure 2A, the area shown is the site below the leading wound edge. The dotted line and asterisk at the top of these images indicate the location of the epithelial leading edge and the dashed line at the bottom of these images indicates the location of the corneal endothelium. Cells beneath the leading edge in Figure 2(Ab) are MPO positive and have similar morphologies to those that stained for β2 integrin in Figure 1(Bii). The bulk of these MPO+ cells were GR1+ but a fraction of the MPO+ cells in the deeper stroma appeared negative for GR1 (Figure 2(Aa,Ac)). The GR1 antibody recognizes members of a family of proteins referred to as Ly-6 proteins including Ly6G and Ly6C. We also stained sections of the injured mouse eyes with antibodies specifically against either Ly6G or Ly6C (Figure 2(Ae–Ag)). The anterior most-immune cells in the cornea were positive for both proteins, with expression levels varying among cells.

To assess the presence of immune cells of the monocyte/macrophage lineage at D1 post wounding, we immunolabeled cryosections with an antibody against CD68 and co-stained with an antibody against Ly6C (Figure 2B). CD68 staining reveals a subpopulation of the immune cells in the corneal stroma at D1. Here we refer to these CD68+ cells as monocytes. These monocytes typically localized below the neutrophils located at the anterior aspect of the corneal stroma. While the majority of CD68+ cells were also Ly6C+ (Figure 2B, arrow), not all Ly6C+ cells were CD68+. Macrophage populations are both diverse and dynamic, and their expression of different molecules can be used to help identify their potential functions. Therefore, we next determined whether the CD68+ cells co-expressed iNOS, a protein characteristic of M1 inflammatory macrophages, or Arg1 and CD206, two proteins characteristic of M2 reparative macrophages. We also assessed the expression F4/80, a marker for a specific subset of mouse tissue macrophages. At D1 post wounding the CD68+ cells did not express any of these molecules (Supplementary Figure S1).

We also localized neutrophils and monocytes in the area in front of the leading edge of the corneal wound that is identified in the schematic presented in Figure 1(Aiii). While neutrophils (Ly6C+/Ly6G+/MPO+) were present under the exposed epithelial basement membrane in the anterior stroma, there were very few CD68+ immune cells, with those present localized to the posterior stroma (Figure 2(Ad,Ah,Bd); Supplementary Figure S2). Quantification of GR1+MPO+ neutrophils and CD68+ cells presented in Figure 2C also corroborates a neutrophil-dominant response shortly after corneal wounding. T cells, assessed using antibodies that identify CD3, CD4, and CD8 populations, were not seen in the corneal stroma at 1D after injury, either under the leading edge or under the exposed stroma (Supplementary Figure S3).

We can conclude that following debridement injury, neutrophils and macrophages are recruited into the stroma below the leading edge during re-epithelialization. In addition, in front of the leading edge, where the epithelial basement membrane and stroma are exposed to the tear film, both neutrophils and monocytes are present. Neutrophils localize to a region immediately below the leading edge in the stroma, where their morphology is elongated and extended; they are also present in the posterior stroma. In contrast, macrophages that had not yet acquired an M2 reparative phenotype appear restricted to the anterior stroma.

### 3.2. Stromal Immune Cells Are Not Detected at D7

We examined whether immune cells remained associated with the corneal stroma at 7 days after debridement, at which time the cornea has re-epithelialized, and the wound is considered healed. Sections from four separate mouse eyes at D7 post wounding were co-immunolabeled for the common immune cell receptor β2 integrin and α6 integrin and co-labeled for F-actin and nuclei. A representative image is presented in Supplementary Figure S4. By this time post injury, the corneal epithelium was intact and restratified. α6 integrin localized to the basement membrane, indicating that hemidesmosomes have partially reformed. While immune cells can be found in the corneal stroma D7 after injury, they were rare (Supplementary Figure S4).

### 3.3. Immune Cells Are Found at Erosion Sites

**Figure 3:**
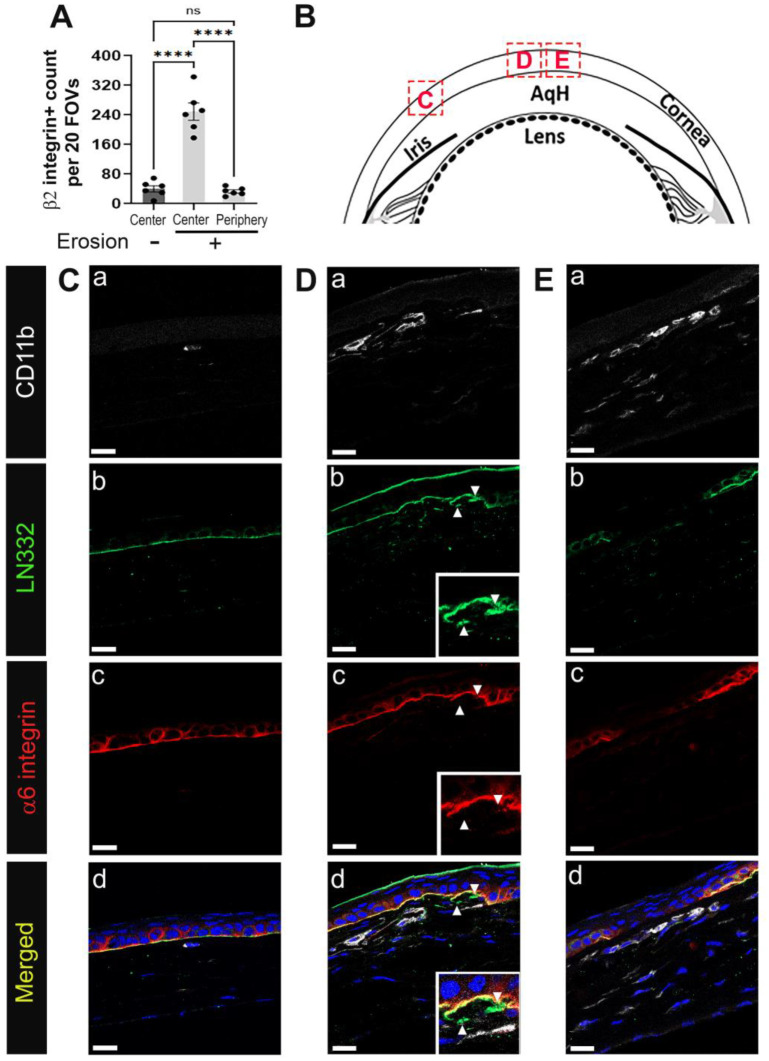
Immune cells are recruited to regions of defective EpBM in developing corneal erosions at D28 post debridement wounding. D28 sections were co-immunolabeled with pan-leukocyte marker β2 integrin or myeloid cell marker CD11b (white) and hemidesmosomal components α6 integrin (red) and LN332 (green). Nuclei were counterstained blue with DAPI. (**A**) Quantification of β2 integrin+ leukocytes in the stroma of corneas with erosion (centrally and peripherally) and without erosion (centrally) (n = 6). Each data point represents the total number of immune cells from 20 FOVs in 4 non-consecutive sections per eye. Barplots show adjusted *p*-value with post-hoc Tukey tests after one-way ANOVA (**** *p*-value < 0.0001). (**B**) Diagram of the cornea with boxed areas indicating regions distal to (**C**) and near the erosion site (**D**,**E**) where shown images were taken. In the eyes of mice with corneal erosions, CD11b+ cells accumulate in the stroma beneath abnormal (**D**) or discontinuous (**E**) BM, but not in regions where the integrity of the EpBM has not been compromised (**C**). Panel **a**–**d** present CD11b, LN332, α6 integrin, and composite staining, respectively, of each boxed region. Insets in (**D**) show higher magnification of perturbed hemidesmosome junction with isolated fragments of LN332 in the stroma (arrowhead). All images are single focal plane and representative of six corneas with erosion. Mag bars: 20 µm. AqH aqueous humor.

To look at the involvement of immune cells in erosion formation, we obtained tissue sections from D28 eyes whose corneas had frank erosions, which was determined by focal staining of a region of the cornea with a vital dye at sacrifice. When evaluating eyes with corneal erosions, sections were obtained distal from the erosion site as well as at sites adjacent to erosions. Figure 3A shows quantification of β2 integrin+ cells in corneas with and without erosions. Corneas lacking erosions had significantly fewer stromal immune cells compared to areas close to the erosion sites, but a comparable number to the periphery of corneas with erosions.

The epithelial layer can appear either intact, dysmorphic, or discontinuous adjacent to an erosion site. In Figure 3B, a schematic is shown highlighting the three sites from which images were acquired for Figure 3C–E. Site C is distal to the erosion, whereas D and E are nearer the erosion site. Tissues were co-immunolabeled with antibodies against α6 integrin, LN332, and the myeloid cell marker CD11b/αM, an integrin that forms heterodimers with β2 integrin. As mentioned above, α6β4 integrin is an epithelial protein that is present in hemidesmosomes and LN332 is its ligand. Together α6β4 integrin and LN332 allow us to assess the integrity of the epithelial basement membrane. Distal to the erosion site (Figure 3(Ca–Cd)), rarely were CD11b positive cells detected. The localization of α6 integrin and LN332 showed that the basement membrane in this region appeared intact and continuous. At site D (Figure 3(Da–Dd)), (arrowheads)) corneal epithelial cells appeared to be invading the stroma or were being pulled into the stroma. Beneath these sites numerous CD11b positive immune cells were present, and these immune cells had elongated morphologies similar to those seen for the neutrophils at D1. At site E (Figure 3(Ea–Ed)), α6 integrin and LN332 were discontinuous and a layer of CD11b+ immune cells was present underneath the epithelium. There were also CD11b+ cells that had localized to the anterior stroma.

**Figure 4:**
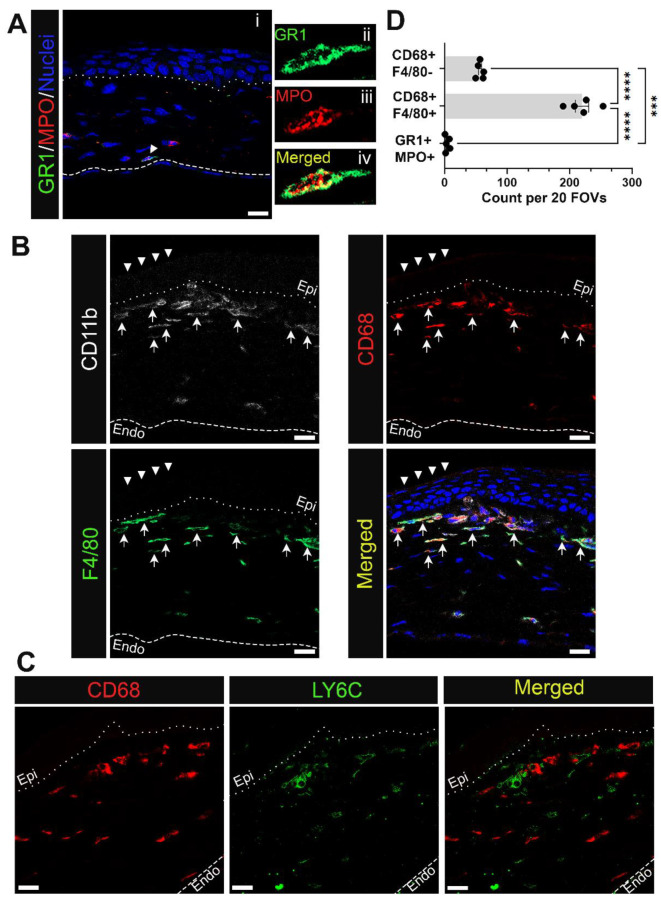
Myeloid cells accumulating in the corneal stroma near erosion sites at D28 post wounding are mostly macrophages and rarely neutrophils. Shown are representative images of immune cells at D28 positive for (**A**) neutrophil markers GR1 (green) and MPO (red) and (**B**) monocyte/macrophage markers CD68 (red) and F4/80 (green) along with pan-myeloid cell marker CD11b (white). Panel (**A**(**ii**–**iv**)) show neutrophil identified in (**A**(**i**)) at higher magnification. Panels in (**B**) show CD68+ F4/80+ cells (arrow) are more concentrated under the normally stratified epithelium (arrowhead) whereas myeloid cells beneath the dysmorphic epithelium display varying expression of CD68 and F4/80. CD68+ cells were assessed further for LY6C expression in (**C**). Neutrophils and subsets of macrophages found near erosion sites were quantified in (**D**) from 20 FOVs in 5 non-consecutive sections per eye (n = 5). Bar plots show adjusted p-value with post-hoc Tukey tests after one-way ANOVA (*** *p* < 0.001, **** *p*-value < 0.0001). Dotted and dashed line represent the EpBM and Descemet’s membrane, respectively. (**A**) 1 µm projection, (**B**,**C**) Single focal plane. Mag bars: 20 µm. Endo corneal endothelium and epi corneal epithelium.

**Figure 5:**
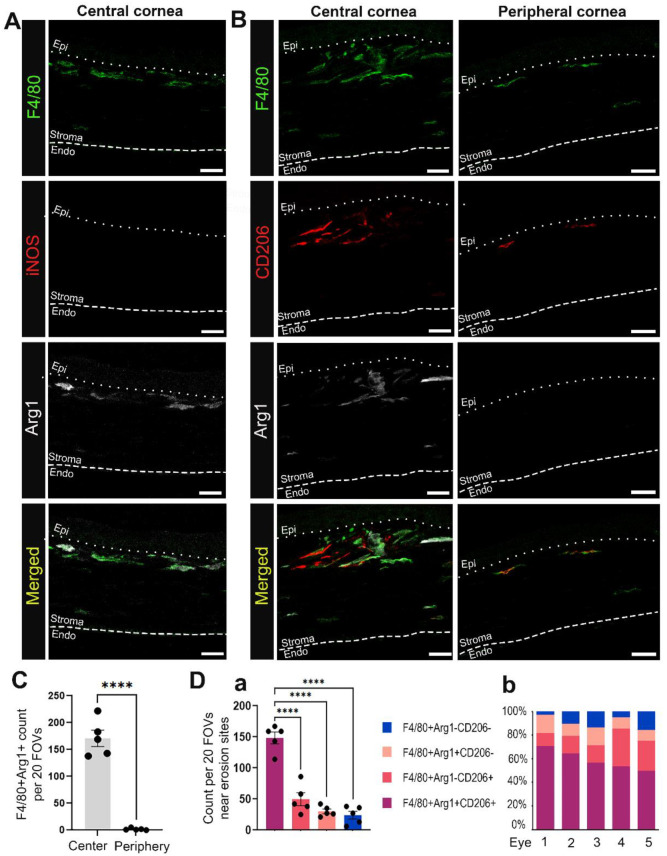
Macrophages migrating into the corneal stroma near erosion sites at D28 post wounding often exhibit an M2 phenotype. Shown are representative images of F4/80+ macrophages (green) at D28 co-labeled with M1 marker iNOS (red) and/or M2 markers including Arg1 (white) and CD206 (red). (**A**) shows the absence of iNOS and presence of Arg1 expression among F4/80+ macrophages in the central cornea of eyes with erosions. (**B**) shows variable expression of M2 phenotypic markers among macrophages recruited to the central and peripheral stroma of corneas with erosion. (**C**) compares the number of F4/80+Arg1+ macrophages found in the central and peripheral cornea. (**Da**) presents quantification of M2 vs. non-M2 subsets near erosion sites in the central cornea. Each datapoint in (**C**,**Da**) represents count from 20 FOVs in 5 non-consecutive sections per eye (n = 5). Bar plots show adjusted *p*-value after *t*-test (**C**) and one-way ANOVA with post-hoc Tukey (**D**) (**** *p*-value < 0.0001). (**Db**) shows percentage contribution of M2 and non-M2 subsets to the total recruited macrophages in the central cornea of each eye examined in (**Da**). All images are 2.5 µm projection. Mag bars: 20 µm. Endo corneal endothelium and epi corneal epithelium.

Since CD11b staining alone doesn’t differentiate between neutrophils, monocytes, and macrophages, sections obtained from corneas with erosions at D28 were immunolabeled using the same complement of antibodies that were used to study immune cells at D1 after debridement wounding. In Figure 4 and Figure 5 the anterior dotted line marks the epithelial-stromal border as determined by DAPI labeling of nuclei. The dashed line in the posterior stroma of Figure 4(Ai) is where Descemet’s membrane and the corneal endothelial nuclei are located. Corneal sections were co-immunolabeled for MPO and GR1 to assess whether any of the immune cells that are present at erosion sites were neutrophils (Figure 4(Ai)). While none of the cells present beneath the epithelium near erosion sites were positive for MPO and GR1, occasional MPO+/GR1+ immune cells were found in the posterior stroma of corneas with erosions (Figure 4(Ai), arrowhead, and shown at higher magnification in Figure 4(Aii–Aiv)). MPO+ immune cells in corneas with erosions were rare as reflected by quantification presented in Figure 4D and were never observed near the corneal epithelium.

Co-immunolabeling studies were also performed in sections containing corneal erosion at D28 after debridement wounding using antibodies to CD11b, CD68, and F4/80 and the nuclear stain DAPI (Figure 4B). Each antibody label is shown both independently and in a merged image together with DAPI labeling. These data show that the CD11b+ myeloid cells present at erosions were CD68+/F4/80+ macrophages (Figure 4B, arrow). The morphologies of these cells varied. On the left side of this image, where the epithelium appeared to be normally stratified (Figure 4B, arrowhead), elongated CD68+/F4/80+ macrophages were organized in multicellular chains. There was heterogeneity to the macrophages present closest to the dysmorphic epithelium. They included CD11b+/CD68+/F4/80^high^, CD11b+/CD68+/F4/80^low^, and to a lesser extent, CD11b+/CD68+/F4/80-immune cells (Figure 4D). The region of the stroma located just below these cells was predominated by CD11b^low^/CD68^low^/F4/80^high^ immune cells. To further characterize the heterogeneity of the immune cells in corneas with erosions, sections were co-immunolabeled for CD68 and Ly6C (Figure 4C). Each antibody label is shown both independently and as a merged image, revealing that CD68+ and Ly6C+ were primarily localized to distinct immune cell populations.

### 3.4. Macrophages in Mouse Corneas with Erosions Express Arg1, CD206, and Not iNOS

**Figure 6:**
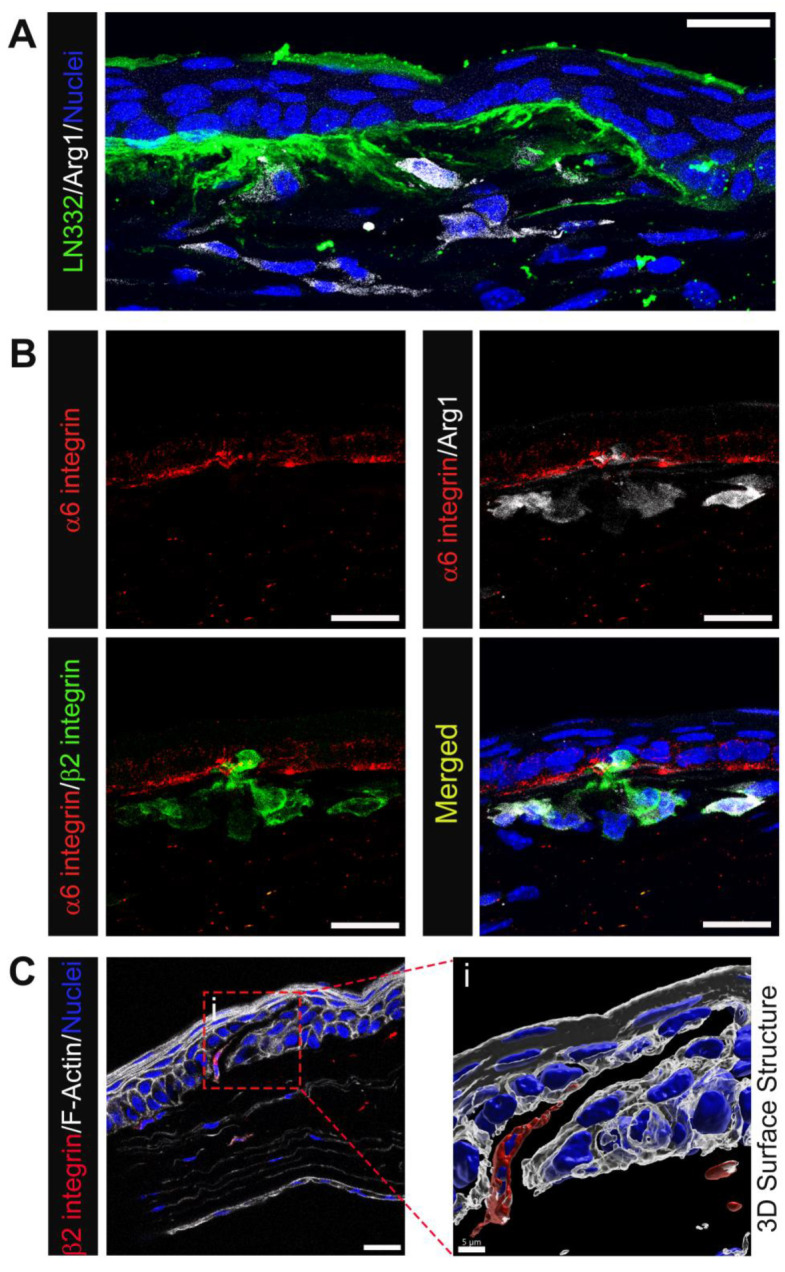
Immune cells, particularly M2-like, populating the corneal stroma at D28 post wounding are closely linked with perturbed EpBM. (**A**) Accumulation of Arg1+ macrophages (white) at sites of abnormal LN332 spill (green) in the central corneal stroma. (**B**) Colocalization of Arg1+β2 integrin+ macrophages (white/green) with disrupted EpBM α6 integrin (red). (**C**) A β2 integrin+ immune cell (red) with migratory morphology populating a crack within the corneal epithelium visualized by phalloidin staining (white). 3D structural rendering of the boxed region in (**C**) with transparent F-actin highlights an area of disconnected epithelial cells between which the immune cell traverses (**Ci**). (**A**,**B**) 1 & 3 µm projection; (**C**) single optical plane; and (**Ci**) 3D surface structure. Mag bars: 20 µm except for (**Ci**) 5 µm.

To determine whether the macrophages present in corneas with erosions at D28 post-debridement wounding were M1 or M2 macrophages, we co-immunolabeled tissues for F4/80, iNOS, and Arg1 (Figure 5A), or F4/80, Arg1, and CD206 (Figure 5B). Figure 5A shows that F4/80+ anterior stromal macrophages were iNOS negative, while a subpopulation were Arg1 positive. Figure 5B–D shows that F4/80+/Arg1+/CD206+, F4/80+/Arg1+/CD206−, F4/80+/Arg1−/CD206+, and F4/80+/Arg1−/CD206− macrophages were all present in the central corneal stroma of the region with erosion in contrast to the peripheral cornea. Many of these macrophages were highly elongated, suggesting that they may be exerting pulling forces on the basement membrane of the epithelium. In Figure 6 we also examined the colocalization of Arg1+ immune cells with α6 integrin and LN332. At sites where Arg1+ cells were present, LN332 was disrupted and spilling into the stroma (Figure 6A). In addition, Arg1+ cells could be seen reaching into the epithelium and disrupting epithelial cell α6 integrin localization (Figure 6B). In Figure 6C, a β2 integrin expressing macrophage is shown migrating into the epithelium and severing connections between epithelial cells.

### 3.5. CD3+ and CD4+ T Cells Can Be Seen at Erosion Sites

**Figure 7:**
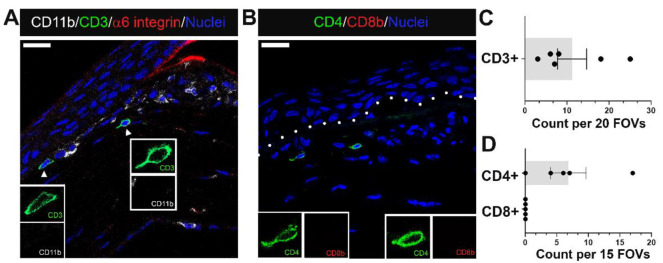
A small population of CD4+ T cells migrate alongside myeloid cells to regions near erosion sites at D28 post debridement wounding. (**A**) shows T cells detected by antibodies against CD3 (green) among immune infiltrate containing mostly CD11b+ myeloid cells (white) in a region where α6 integrin (red) of EpBM is disrupted. (**B**) identifies recruited T cells as CD4+ T helper cells (green), and not CD8+ cytotoxic T (red) cells. Quantification of CD3+, CD4+, and CD8+ T cells in the central cornea of eyes with erosion examined is presented in (**C**,**D**). Each data point represents the count in 20 FOVs for (**C**) (n = 6) and 15 FOVs for (**D**) (n = 5). Insets represent higher magnification of CD11b and CD3 (**A**), or CD4 and CD8b (**B**) expression of migrating T cells. Nuclei were counterstained blue with DAPI. Images are representative of five corneas. Mag bars: 20 µm. Dotted line delineates EpBM.

In addition to neutrophils, monocytes, and macrophages, we have examined whether T cell populations associated with the stroma of corneas with erosions. Sections were co-immunolabeled for the T cell antigen CD3, the myeloid cell receptor CD11b/αM integrin and α6 integrin (Figure 7A). CD11b and α6 integrin staining confirmed that the basement membrane was disrupted in this cornea and that there were numerous CD11b+ macrophages. A few CD3+ immune cells were detected at sites where erosions were present and the basement membrane disrupted (Figure 7A,C). CD3 is a surface protein expressed on naive T cells that functions, along with other proteins, to activate expression of CD4 on T-helper cells and CD8 on cytotoxic T cells. Therefore, we also co-immunolabeled corneas with erosions for CD4 and CD8 (Figure 7B). The results show that the CD3+ T cells detected were CD4+ and CD8− (Figure 7B,D). These results indicate that when present at erosion sites, T cells were not cytotoxic T cells. We have not yet determined whether there were other types of T cells present.

### 3.6. Whether or Not Erosions Develop, Corneal Sensory Nerve Reinnervation Remains Partial at D28 after Debridement Injury

**Figure 8:**
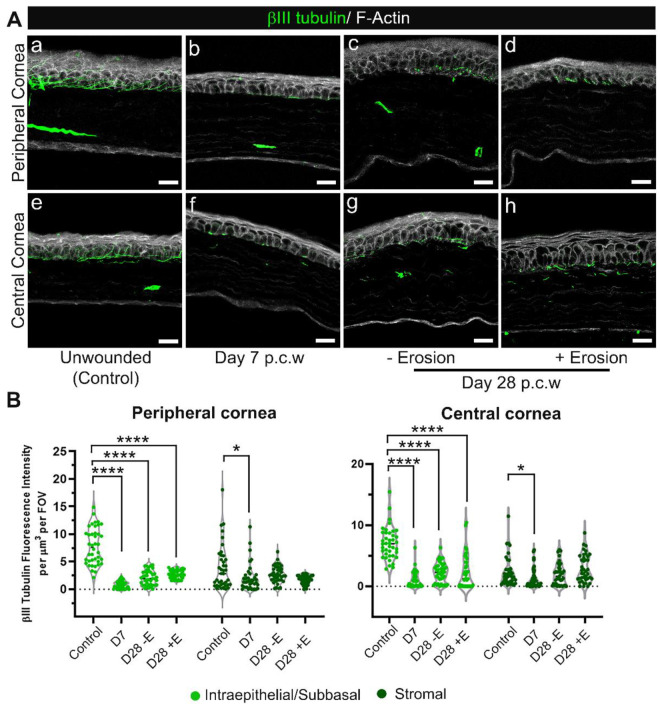
Reinnervation of the corneal epithelium remains sparse at D28 post debridement wounding regardless of erosion formation. Sections from unwounded and wounded corneas at D7 and D28 (+E and −E, i.e., with and without erosion formation) post corneal wounding (p.c.w) were stained with βIII tubulin (green) and phalloidin (white) to visualize distribution of stromal nerves, sub-basal nerve plexus and intraepithelial nerve terminals. (**A**) Shown are representative images from both the peripheral (**a**–**d**) and central (**e**–**h**) cornea in specific conditions listed above. Five corneas per condition were examined. (**B**) Quantification of nerve presence in 40 FOVs, either peripheral (**left**) or central (**right**), taken from 4 corneas per condition. Nerve tracing was performed by 3D surface rendering using Imaris. Each data point is cumulative βIII tubulin fluorescence intensity obtained from specified region, either intraepithelial/sub-basal or stromal, of each Z-stack divided by the volume of the same region. Violin plots show adjusted *p*-value from pairwise comparisons against the unwounded (control) after Kruskal–Wallis tests (*p* < 0.0001 for the intraepithelial-sun-basal region & *p* < 0.01 for the stromal region of the cornea) (* *p* < 0.05, **** *p* < 0.0001). (**Aa**,**e**) 2.5 µm projection (**Ab**–**Ad**), and (**Af**–**Ah**) 5–7.5 µm projection. Mag bars: 20 µm.

Inflammation has been implicated in the loss of corneal nerves in autoimmune and dry eye disease [22,23]. Since we showed in Figure 3A that corneas with erosions had significantly more immune cells in their stroma compared to corneas without erosions, we next asked whether eyes with erosions showed differences in their corneal nerves compared to those without erosions. Next, we immunolabelled unwounded D7 and D28 corneas with and without erosions using an anti-βIII tubulin antibody and co-stained for F-actin (Figure 8). The intraepithelial corneal sensory nerves are shown in both central and peripheral regions. The intraepithelial corneal nerves in controls (Figure 8(Aa,Ae)) extended parallel to the epithelial basement membrane and branched apically towards the apical squames where tight junctions are located. In Figure 8(Ab–Ad,Af–Ah), we observed denervation persisting both in the periphery and at the center of the cornea at D7 and D28, whether or not erosions had formed. Quantification of βIII tubulin staining across different timepoints further substantiated incomplete re-innervation at the intraepithelial/sub-basal level even though some recovery could be observed from the larger spread of data points over time (Figure 8B). The presence of stromal nerves on the other hand was significantly different from controls only at D7 but not at D28 post wounding, whether or not or erosions were present.

## 4. Discussion

**Figure 9:**
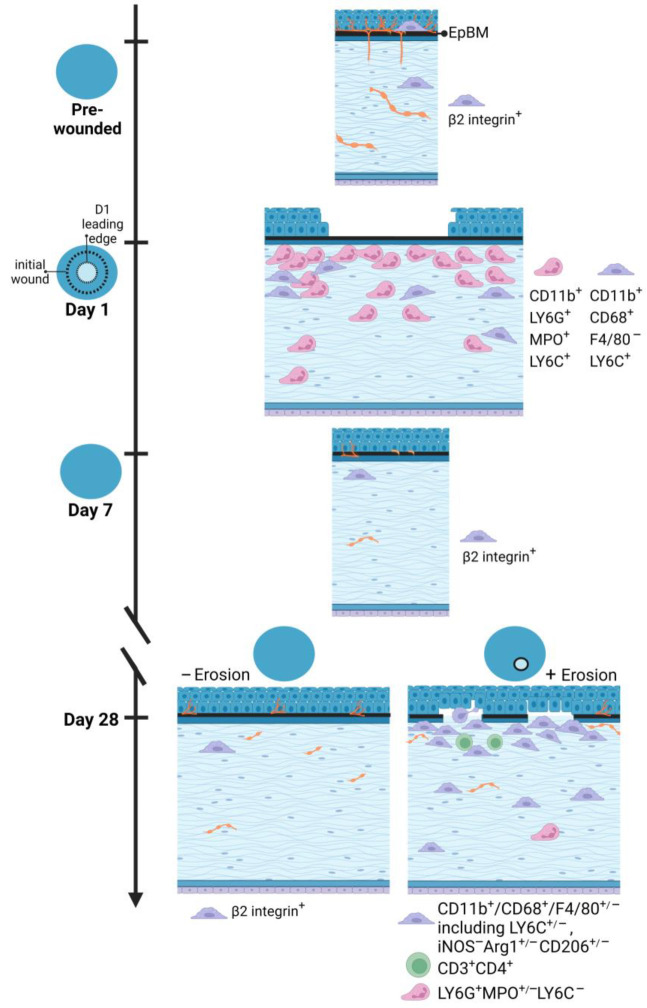
An illustrated summary of immune cell recruitment and corneal re-innervation state in response to corneal debridement wounding versus erosion formation. Data presented show a differential immune response to shortly after debridement injury compared to the spontaneous development of corneal erosions post wounding. While the former is heavily neutrophilic, the latter is dominated by M2 macrophages with adaptive immunity involved. Innervation of the corneal epithelium is not fully re-established even at D28 after wounding regardless of erosion status. Model created with BioRender (https://www.biorender.com/).

The model presented in Figure 9 summarizes the results described in this study. It takes several weeks for hemidesmosomes to reform and restore the stability of the ocular surface after debridement injury. At D1 neutrophils and monocytes are present under the leading edge and in the bare stroma. Six days after the epithelial barrier had been restored, at D7, few immune cells remain in the central cornea; by this time point, immune cells recruited into the cornea at D1 have either undergone apoptosis or migrated to local lymph nodes. The number of resident immune cells returns, over time, to control levels. Our results suggest that this return to normal numbers of resident immune cells takes longer than 7 days.

During this recovery period the ocular surface remains at risk of erosion formation. In people, recurrent erosions can form secondary to eye rubbing [24,25]. They also form with increased frequency in diabetics, after viral infections, in patients with basement membrane dystrophies, and in the elderly [1,2,3]. In the mouse debridement injury model, we have shown that erosion formation occurs secondary to persistently elevated epithelial cell proliferation and that erosion frequency can be reduced by treating the cornea with mitomycin C at the time of injury [7,26,27].

The failure of the sensory nerves to fully reinnervate the C57BL/6 mouse cornea was not surprising given that we have previously reported that erosion formation interferes with nerve re-growth in BALB/c mice [14]. Here we show that D28 corneas with and without erosions at the time of euthanasia had similar axon densities despite having different numbers of stromal immune cells. These data suggest that delayed reinnervation is not caused by chronic inflammation and support our previous data showing that suppressing epithelial cell proliferation using a single treatment with mitomycin C at the time of debridement injury reduces erosion formation [7,27].

The erosions seen in this model are recurrent [28]; they are not ulcers where the epithelium never closes the wound site. The first signs of pathology appear at D14 as cells at developing erosions cease proliferating and begin to undergo senescence [26]. Erosions present at D14 resolve spontaneously, and by D21 and D28 erosions will either disappear entirely or reappear at different sites in the same cornea [28]. In this study, we now show that erosions at D28 lacked MPO+ neutrophils at the epithelial–stromal interface. Despite recurrent erosions exposing the stroma to the tear film and inducing epithelial cell migration, here our studies show for the first time that recurrent erosions do not initiate a de novo innate immune response involving neutrophils. Instead, we find that there were numerous Arg1+ CD206+ macrophages associated with the occurrence of corneal erosions, both of which are features of M2 macrophages. We find no M1 macrophages present in this wound model at erosion sites; M1 macrophages are induced by microbial stimuli which are present at low levels in this sterile debridement wound model. M2 macrophage phenotype is known to produce a variety of anti-inflammatory mediators and growth factors that suppress inflammation, as well as to promote wound healing and tissue repair [29]. Arg1 also has been shown to play a key role in matrix deposition at wound sites [30]. Though M2 recruitment has been recognized in several corneal wounding models [31,32,33,34], our studies provide the first evidence of their presence in the context of recurrent erosions. A spatial link between this reparative macrophage phenotype and regions of perturbed epithelial basement membrane as shown in this study suggests a potential role for M2 macrophages in erosion repair.

Immune memory was first recognized by the ancient Greeks and is known to be responsible for providing long-term protection from pathogens and for the efficacy of vaccines [35,36]. The immune cells that are responsible for immune memory have been defined functionally as long-lived cells that are maintained independently of stimulation during homeostasis or by long-term maintenance. This classic definition of memory immune cells requires the cell to be specific for a given epitope. Memory immune cells should also be intrinsically altered by their previous encounter with antigens, allowing them to respond more quickly to subsequent encounters with a specific pathogen or antigen [36]. Epigenetics provides a potential mechanism for maintaining immune memory. Memory is a vital component of the adaptive immune system and has been best characterized in memory T cells [37]. Memory involves the T cells secreting specific cytokines that function to prime resident cells to respond more rapidly to subsequent epitope exposure.

While immune memory in response to infections has been studied for hundreds of years, how immune memory functions in tissue injury repair is less clear. The corneal epithelium, like other surface epithelia including the skin, gut, and airway epithelia, are subject to continuous assaults, most minor but some major. Prior injury has been shown to increase the rate of re-epithelialization and barrier restoration in the cornea [38], skin [39], lungs [40], and intestine [41]. While epigenetics [42] and increased cytokine expression [38] have been implicated in regulating these events, whether specific immune cell types or epigenetic memory in the epithelial cells themselves play roles remains an open question. Here we show that when corneal erosions are recurrent, the re-exposure of the stroma to the tear film does not trigger neutrophil influx as seen in D1 after injury. These data in the C57BL/6 mouse cornea suggest that tissue memory derived from prior injury protects the cornea from rupturing under conditions when the cornea is subjected to recurrent epithelial defects. Future studies are needed to confirm whether the few CD3+ and CD4+ T cells present in corneas with erosions are memory T cells or whether, instead, it is the corneal epithelial cells and/or sensory neurons that play these important roles.

## Figures and Tables

**Table 1:** Primary antibodies.

Antibody	Clone	Dilution	Host	Company	Conjugate	Cat #
Arginase1	D4E3M	1:50	Rabbit	Cell Signaling	Alexa Fluor 488 & 647	66297S & 43279S
β2 integrin	M18/2	1:50	Rat	Biolegend	Alexa Fluor 594	101416
β3 tubulin	N/A	1:100	Rabbit	Abcam	N/A	ab18207
CD11b	M1/70	1:200	Rat	Abcam	Alexa Fluor 647	ab197702
CD206	C068C2	1:50	Rat	Biolegend	Alexa Fluor 647	141712
CD3	17A2	1:100	Rat	Biolegend	Alexa Fluor 488	100210
CD4	RM4-5	1:100	Rat	Biolegend	Alexa Fluor 488	100532
CD49f/ α6 integrin	GoH3	1:100	Rat	BD Pharmingen	N/A	555734
CD68	FA-11	1:50	Rat	Biolegend	Alexa Fluor 488 & 594	137012 & 137020
CD8b	YTS156.7.7	1:50	Rat	Biolegend	Alexa Fluor 594	126635
F4/80	BM8	1:50	Rat	Biolegend	Alexa Fluor 488 & 594	123120 & 123140
GR1	RB6-8C5	1:50	Rat	Stem Cell Technologies	Alexa Fluor 488	60028AD
iNOS	D6B6S	1:50	Rabbit	Cell Signaling	Alexa Fluor 488	93421S
LMN332	J18	1:100	Rabbit	Jonathan C. Jones, Kevin Hamill [20]		
LY6C	ER-MP20	1:100	Rat	Invitrogen	N/A	MA1-81899
LY6G	1A8	1:100	Rat	Biolegend	Alexa Fluor 488	127626
Myeloperoxidase	N/A	1:100	Goat	R&D Systems	N/A	AF3667

**Table 2:** Secondary antibodies.

Host	Target	Conjugate	Jackson ImmunoResearch Cat #
Donkey	Goat	Alexa Fluor 594	705-585-003
Goat	Rat	Alexa Fluor 594	112-585-003
Goat	Rat	Alexa Fluor 647	112-605-003
Goat	Rat	Alexa Fluor 488	112-545-167
Goat	Rabbit	Alexa Fluor 488	111-545-003

## Data Availability

Not applicable.

## Supplementary Materials

The following supporting information can be downloaded at: https://www.mdpi.com/article/10.3390/biom13071059/s1, Figure S1: CD68+ monocytes/macrophages infiltrating the central corneal stroma 1 day post debridement wounding neither express F4/80 nor exhibit M1/M2 polarization; Figure S2: The stroma beneath the open wound 1 day post debridement wounding is mainly populated by neutrophils and rarely by CD68+ monocytes/macrophages; Figure S3: No T cells is detected in the central stroma 1 day post debridement wounding; Figure S4: The frequency of immune cells in the corneal stroma significantly reduces by day 7 post debridement wounding.
