# Supplementary material for: Immune Cells Localize to Sites of Corneal Erosions in C57BL/6 Mice
Source: Biomolecules. 2023 Jun 29;13(7):1059. doi: 10.3390/biom13071059 (PMC10377654; doi:10.3390/biom13071059)
Supplement: Supplementary file 1 [file biomolecules-13-01059-s001.zip › biomolecules-2440116-supplementary.pdf]

Supplementary Materials for

Immune cells localize to sites of corneal erosions in C57BL/6 mice

Phuong M. Le[1], Sonali Pal-Ghosh[2], A. Sue Menko[1], and Mary Ann Stepp[2,3]*

This PDF file includes Supplemental Figures S1 to S4

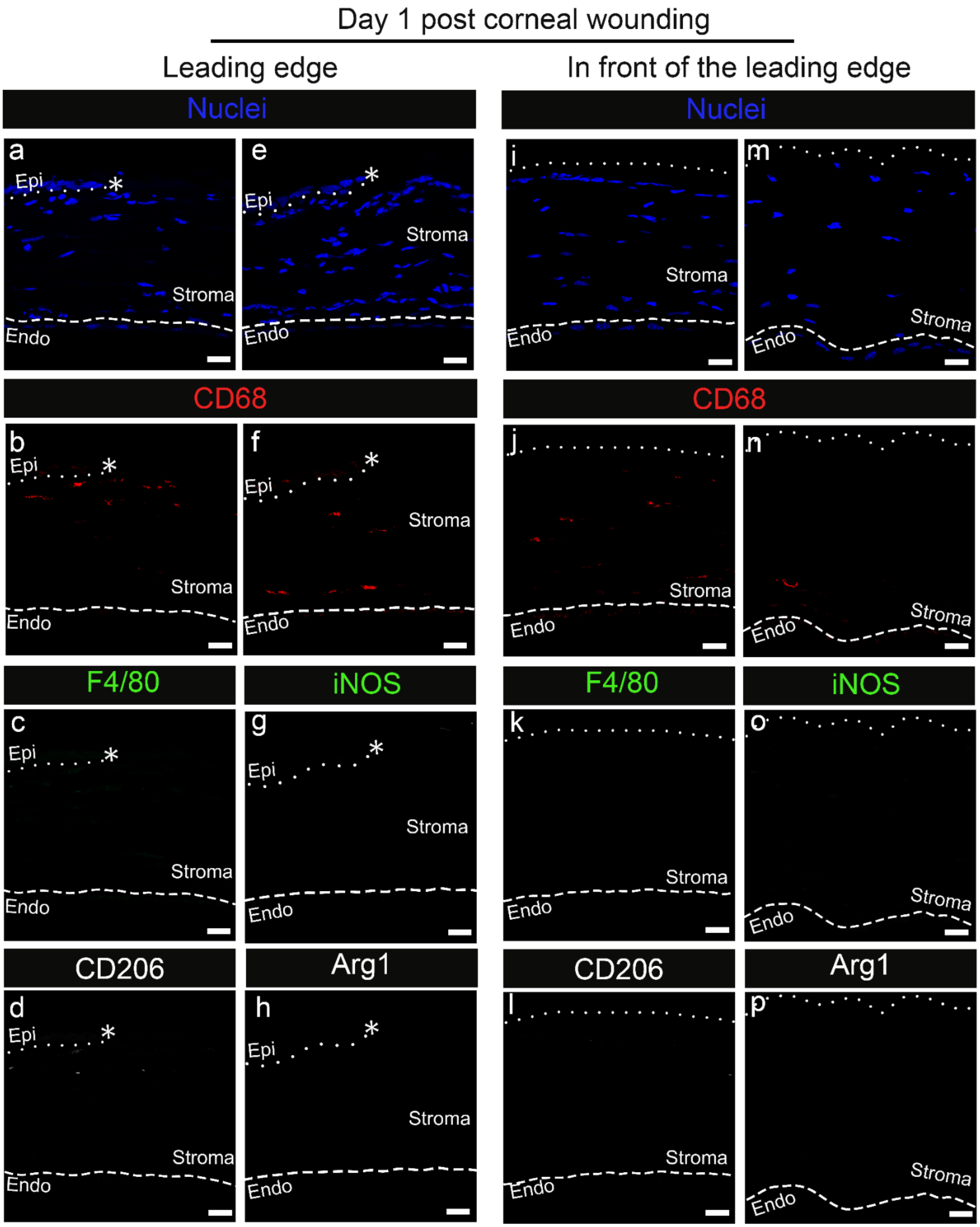

Supplemental Figure S1. CD68+ monocytes/macrophages infiltrating the central corneal stroma 1 day post debridement wounding neither express F4/80 nor exhibit M1/M2 polarization. Shown are representative images of CD68+ cells labeled with F4/80 (green), M1 marker iNOS (green), and M2 markers (CD206, Arg1) (white) in regions beneath the leading edge (a-h) and in front of the leading edge (i-p). Nuclei were stained with DAPI (blue). Asterisk, dotted line, and dashed line represent the wound edge, EpBM, Descemet's membrane, respectively. All images are 5μm projection. Mag bars: 20μm. Endo corneal endothelium, Epi corneal epithelium.

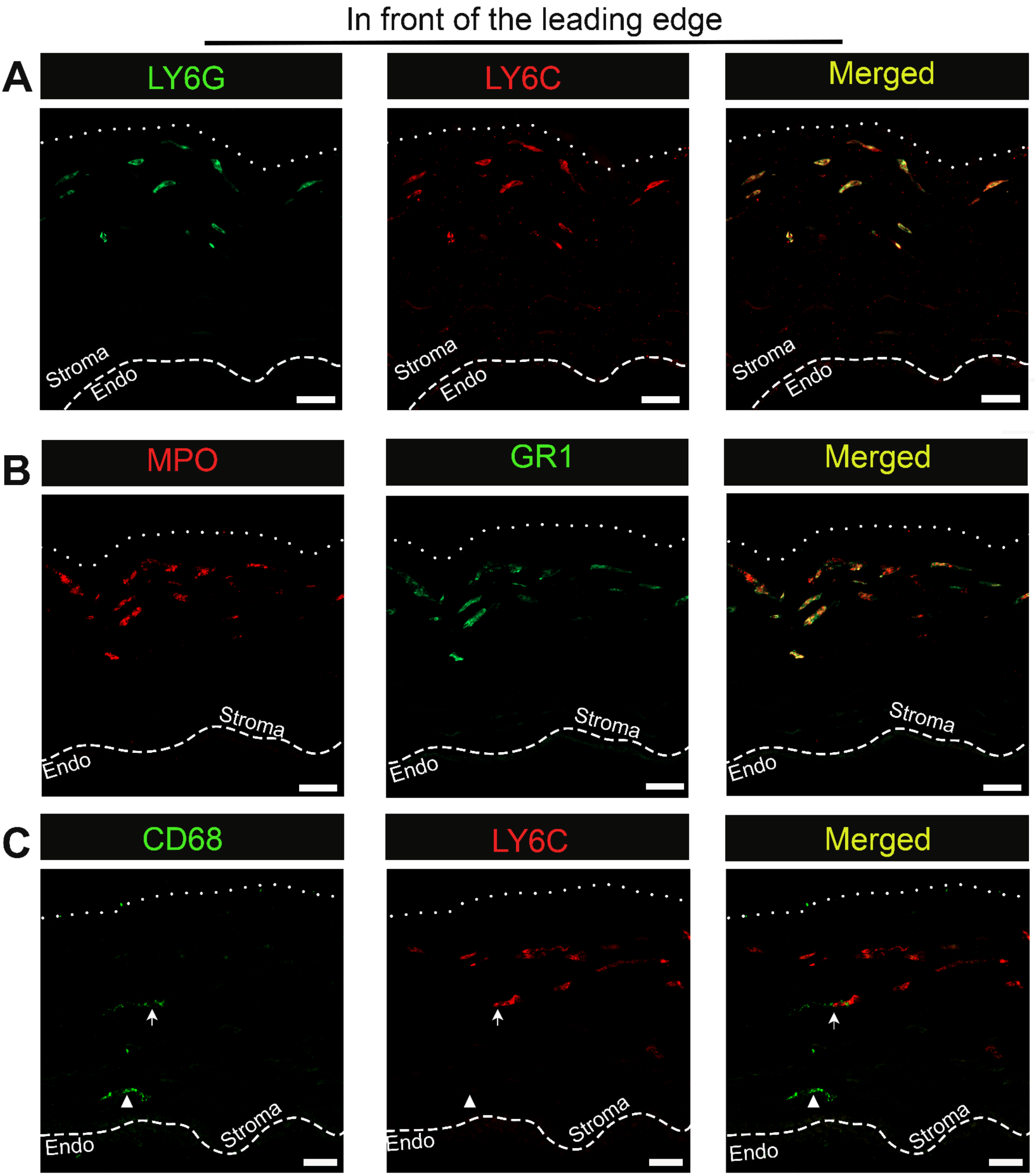

Supplemental Figure S2. The stroma beneath the open wound 1 day post debridement wounding is mainly populated by neutrophils and rarely by CD68+ monocytes/macrophages. Representative images of neutrophils co-expressing (A) LY6G (green) and LY6C (red) or (B) GR1 (green) and MPO (red). Please note that anti-GR1 (green) recognizes a shared epitope of LY6C and LY6G. (C) Representative images of CD68+ macrophages that can be either LY6C+ (arrow) or LY6C- (arrowhead). Dotted and dashed line represent the EpBM and Descemet's membrane, respectively. (A) 3µm projection, (B) 5µm projection. Mag bars: 20µm. Endo corneal endothelium.

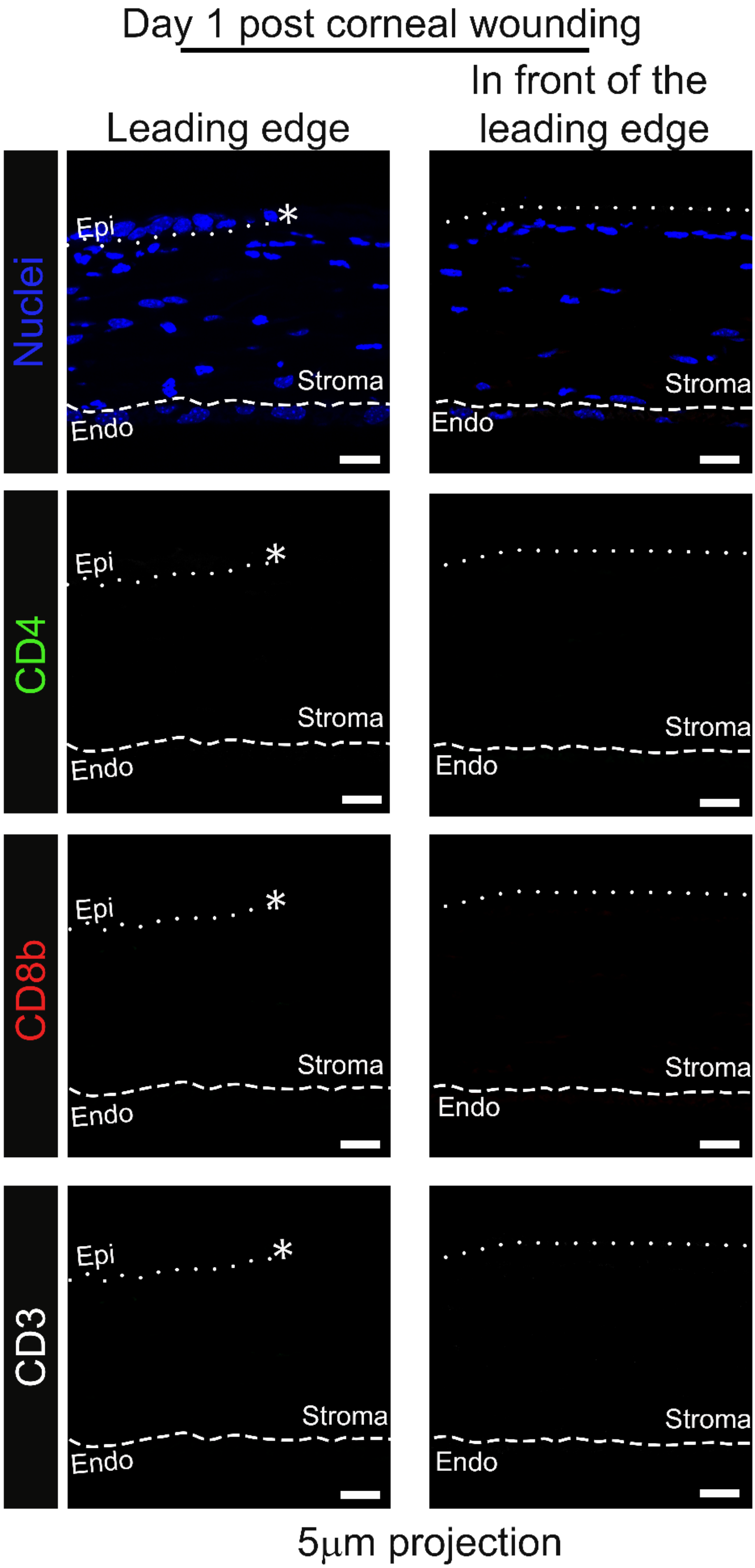

Supplemental Figure S3.
No T cells is detected in the central corneal stroma 1 day post debridement wounding. Representative images of the stromal regions beneath the leading edge (left column) and in front of the leading edge (right column) co-labeled with T cell markers (CD3, CD4, CD8 β chain). Nuclei were stained with DAPI (blue). Asterisk, dotted line, and dashed line represent the wound edge, EpBM, Descemet's membrane, respectively. All images are 5μm projection. Mag bars: 20μm. Endo corneal endothelium, Epi corneal epithelium.

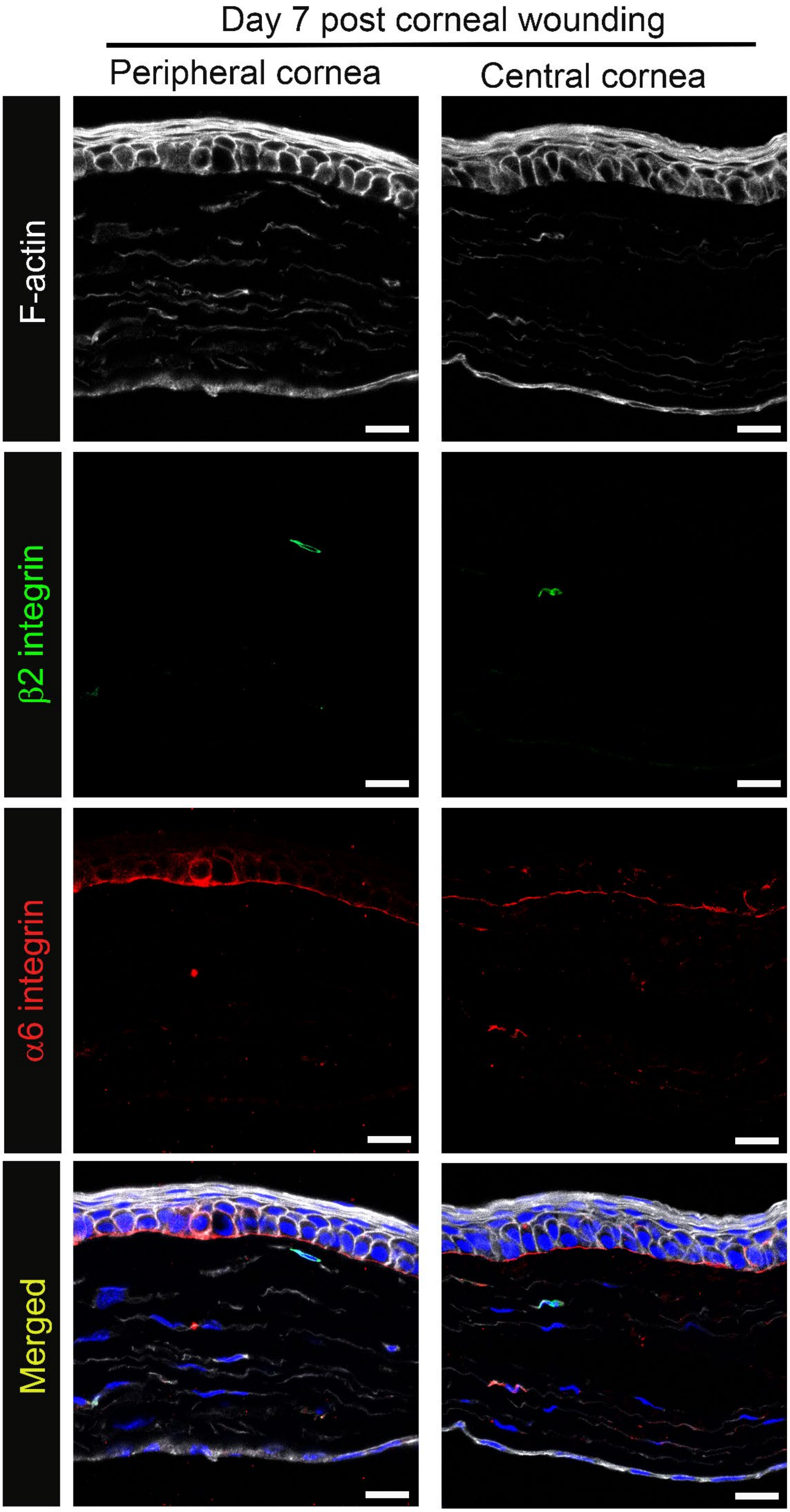

Supplemental Figure S4.
The frequency of immune cells in the corneal stroma significantly reduces by day 7 post debridement wounding. Day 7 sections were co-labeled with β2 integrin (green) and phalloidin (white) to visualize reepithelialization and cellular components in the corneal stroma beneath the EpBM that consists of α6 integrin (red). Nuclei were counterstained blue with DAPI. Shown are representative 7.5 μm projection images of the peripheral cornea (left column) and central cornea (right column) from four wounded corneas. Mag bars: 20μm.
